# Endothelin-1 supports clonal derivation and expansion of cardiovascular progenitors derived from human embryonic stem cells

**DOI:** 10.1038/ncomms10774

**Published:** 2016-03-08

**Authors:** Boon-Seng Soh, Shi-Yan Ng, Hao Wu, Kristina Buac, Joo-Hye C. Park, Xiaojun Lian, Jiejia Xu, Kylie S. Foo, Ulrika Felldin, Xiaobing He, Massimo Nichane, Henry Yang, Lei Bu, Ronald A. Li, Bing Lim, Kenneth R. Chien

**Affiliations:** 1 Cardiovascular Research Center, Massachusetts General Hospital, 185 Cambridge Street, Boston, Massachusetts 02114, USA; 2 Department of Stem Cell and Regenerative Biology, Harvard University, 7 Divinity Avenue, Cambridge, Massachusetts 02138, USA; 3 Li Dak-Sum Research Centre - HKU-Karolinska Institutet Collaboration on Regenerative Medicine, University of Hong Kong, Hong Kong, China; 4 Lau Ming-Wai Center for Regenerative Medicine, Karolinska Institutet, Hong Kong, China; 5 Department of Cell and Molecular Biology and Medicine, Karolinska Institute, Stockholm S-171 77, Sweden; 6 Department of Biochemistry and Molecular Biology, University of Georgia, Athens, Georgia 30602, USA; 7 Stem Cell and Developmental Biology, Genome Institute of Singapore, 60 Biopolis Street #02-01, Genome, Singapore 138672, Singapore; 8 Cancer Institute of Singapore, National University of Singapore, 28 Medical Drive, Singapore 117456, Singapore

## Abstract

Coronary arteriogenesis is a central step in cardiogenesis, requiring coordinated generation and integration of endothelial cell and vascular smooth muscle cells. At present, it is unclear whether the cell fate programme of cardiac progenitors to generate complex muscular or vascular structures is entirely cell autonomous. Here we demonstrate the intrinsic ability of vascular progenitors to develop and self-organize into cardiac tissues by clonally isolating and expanding second heart field cardiovascular progenitors using WNT3A and endothelin-1 (EDN1) human recombinant proteins. Progenitor clones undergo long-term expansion and differentiate primarily into endothelial and smooth muscle cell lineages *in vitro*, and contribute extensively to coronary-like vessels *in vivo*, forming a functional human–mouse chimeric circulatory system. Our study identifies EDN1 as a key factor towards the generation and clonal derivation of ISL1^+^ vascular intermediates, and demonstrates the intrinsic cell-autonomous nature of these progenitors to differentiate and self-organize into functional vasculatures *in vivo*.

## Abstract (web summary)

Understanding coronary vessels development provides basis for regenerative strategies. Here, Soh *et al.* identify endothelin-1 as a key molecule driving long-term expansion of ISL1^+^ bipotent vascular progenitors derived from human embryonic stem cells, and show that these cells can regenerate coronary vessels in mice.

## Graphical abstract

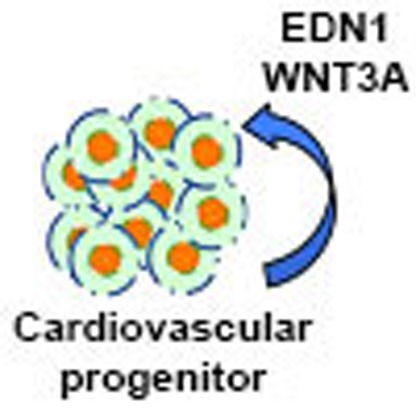

The heart is one of the first organs to be formed in the embryo, and its function is critical for the circulation of nutrients and the removal of waste products to ensure proper development of the embryo subsequently. The formation of the mammalian heart is a complex process, involving two distinct groups of mesodermal progenitor cells, the first and second heart fields (FHF and SHF, respectively). While the FHF primarily gives rise to the left ventricle and parts of atria, the SHF develops into the right ventricle, outflow tract (OFT) and parts of atria^1^,^2^,^3^. Both FHF and SHF cardiac progenitors, marked by HCN4 and ISL1, respectively, have since been isolated and characterized^4^,^5^,^6^,^7^.

Unlike pluripotent stem cells (embryonic or induced pluripotent stem cells), where culture systems have been well-studied and defined culture conditions have been robustly established^8^,^9^,^10^, only a few adult progenitor stem cell populations such as the haematopoietic stem cells, pancreatic progenitors and bone-marrow-derived human mesenchymal stem cells (hMSCs) are able to propagate long-term in defined conditions^11^,^12^,^13^. Other studies reporting the expansion of committed endocrine progenitors, and endoderm progenitors derived from embryonic stem cells (ESCs) or induced pluriportent stem cells also require the co-culture with organ-matched mesenchyme or mouse embryonic fibroblasts (MEFs), respectively^14^,^15^. At present, ISL1^+^ SHF cardiovascular progenitors (CVPs) can only be expanded and kept multipotent on WNT3A-over-expressing feeders^16^. Owing to a lack of robust, defined long-term culture conditions that is capable of maintaining clonally expanded, multipotent ISL1^+^ CVP over prolong periods, we sought to identify novel factors that can support the long-term propagation of ISL1^+^ CVPs *in vitro*.

To achieve this, we differentiated human ESCs with ISL1^+^ reporter into CVPs, and adopted a comparative bioinformatics approach to identify candidate signalling pathways that may be active in uncommitted ISL1^+^ CVPs. This was achieved by performing microarray analysis on subregions of the fetal heart, followed by pairing the paracrine factors present in specific regions, with corresponding receptors that are highly expressed in uncommitted CVPs. The rationale of this approach is that during early cardiogenesis, it is the surrounding mesenchyme that secretes factors to either maintain the proliferation of progenitor cells or provide signals to direct their differentiation. Therefore, examination of paracrine factors that are specific and highly expressed in both OFT and right ventricle would be useful for clonal expansion of ISL1^+^ CVPs that were previously shown to be specifically present in these two regions of the heart. In our study, we identified new factors that support CVP expansion and maintenance, as well as major pathways previously reported to be important through knockout mice models, indicating that our approach was successful. Testing our top candidate endothelin-1 (EDN1), we demonstrated that the factor supports clonal expansion of self-organizing human ISL1^+^ CVPs in a defined culture condition.

## Results

### Growth factor-receptor pairs enriched in the SHF progenitors

To begin addressing the self-assembly ability of CVPs, we first demonstrated clonal derivation of SHF multipotent progenitors. Taking a genomic approach, we identified EDN1 as a potential growth factor that maintains clonal expansion of CVPs in a chemically defined, serum-free medium. Using previously curated list of growth factors and growth factor receptors expressed in humans^8^, and applying the criteria of signal intensity ≥20 from the Illumina array data (background signal subtracted), several hundreds of growth factors expressed in different regions (two atria, two ventricles and OFT) of 11-week-old human embryonic fetal hearts were identified (Supplementary Data 1–5). Similarly, a database for membrane receptors expressed in multipotent ISL1^+^ CVPs was obtained through florescence-activated cell sorting of differentiated human ESCs at day 7 using an established protocol^4^ (Supplementary Fig. 1a). This was achieved using an *ISL1*-Cre knock-in human ESC line (H9G1) previously generated in our laboratory^4^. ISL1^+^ CVPs were isolated by sorting for ISL1^+^/CD24^−^/Pan-Neuronal^−^ population, thus eliminating subsets of ISL1^+^ cells belonging to the pancreatic islet and neuronal lineages^17^,^18^ (Supplementary Fig. 1b).

To identify receptors in CVPs that may be involved in their maintenance, we further imposed stricter criteria of signal intensity ≥50 and day 7 ISL1^+^ cells/day 14 ISL1^+^-derived endothelial cell ratio ≥1.2 to select for receptors that are significantly downregulated when multipotent cardiac progenitors differentiate. A total of 57 membrane receptors were identified (Supplementary Data 6). Further analysis using interaction data from Biomolecular Interaction Network Database (BIND) and Human Protein Reference Database (HPRD) to examine useful ligand–receptor interactions utilized by ISL1^+^ CVPs to maintain multipotency revealed 11 interaction growth factor-growth factor receptor pairs (GF-GFR), involving eight growth factors that are highly expressed in both OFT and right ventricle of the human fetal heart (Supplementary Data 7 and Supplementary Fig. 1c). Not surprisingly, all the identified growth factors have been previously shown to manifest cardiovascular-related phenotypes in knockout mouse models (Supplementary Data 7). From our analysis, EDN1 emerged as the top candidate that can potentially maintain and expand CVP.

### Endothelin-1 supports multipotency of SHF CVP

**Figure 1:**
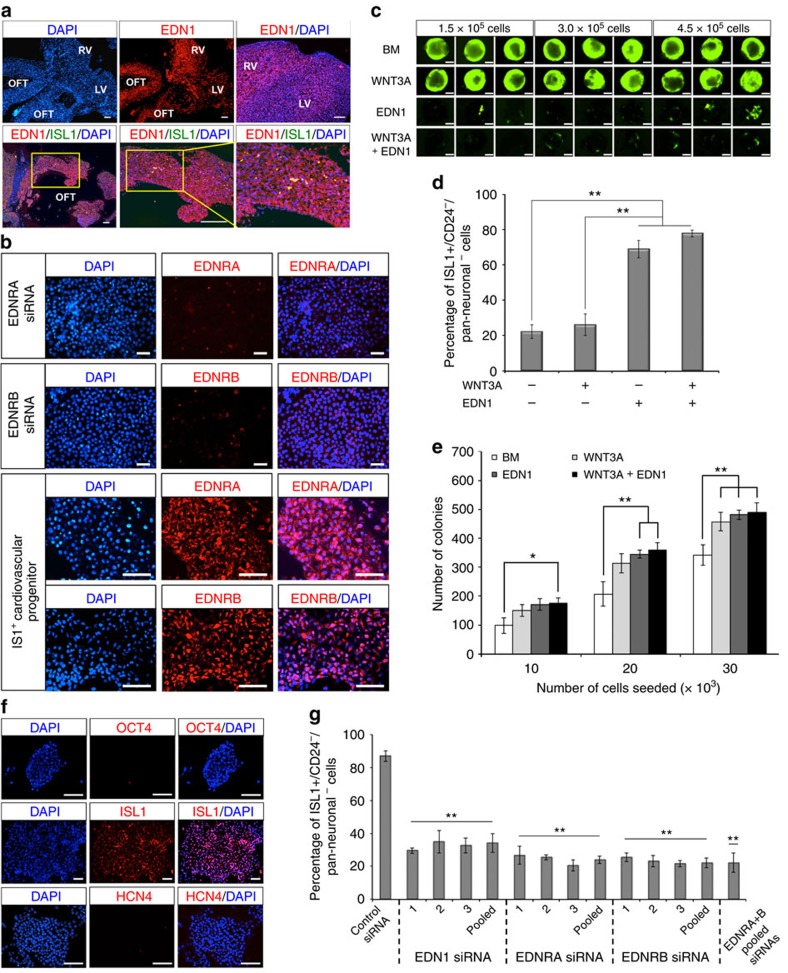
Endothelin-1 supports clonal isolation of ISL1^+^ CVP. (**a**) Immunofluorescence on week 11 human fetal heart shows strong expression of EDN1 (stained red) at both OFT and right ventricle (RV). Enlarged images (indicated by yellow boxes) show small clusters of ISL1^+^ CVPs in the walls of OFT. *n*=3 experiments. Scale bars, 100 μm. (**b**) Immunocytochemistry demonstrating high expressions of EDNRA and EDNRB receptors (stained red) for EDN1 in ISL1^+^ CVPs. EDNRA and EDNRB staining in siRNA-treated ISL1^+^ CVPs serves as negative control. Cell nuclei (blue) were stained with 4,6-diamindino-2-phenylindole (DAPI). Scale bar, 50 μm. (**c**) Differentiation of human ESCs (H9G1) towards *ISL1*-expressing cells (GFP^+^) in embryoid bodies (EBs) when cultured in basal medium (BM) supplemented with 100 ng ml^−1^ EDN1, 100 ng ml^−1^ WNT3A or both. Low numbers of GFP^+^ cells were observed in various EB sizes (1.5 × 10^5^, 3.0 × 10^5^ and 4.5 × 10^5^ cells) cultured in the presence of EDN1. Scale bars, 0.5 mm. (**d**) Flow cytometric analysis of ISL1^+^ cells in EBs. Human ESCs differentiated in media containing EDN1 showed high percentages (≥70%) of cells, which were negative for both CD24 and Pan-Neuronal surface antigens. Error bars indicate s.d., *n*=3 experiments. **P<0.001, evaluated by Student's *t*-test. (**e**) Graphical representation of colony-forming efficiency of the ISL1^+^ CVPs in BM supplemented with WNT3A, EDN1 or both. Single-progenitor cells were seeded on MEF feeders at various cell densities (10,000, 20,000 and 30,000 cells) per well in six-well plates. Colonies were visible after day 7 and were subsequently stained with crystal violet for quantitation. Error bars indicate s.d., *n*=3 experiments. *P=0.05 and **P<0.03, evaluated by Student's *t*-test. (**f**) Immunocytochemistry illustrating the expression of *ISL1* in H9G1-derived CVP clones. The expression of *ISL1* gene (red) was revealed as a nuclear protein. ISL^+^ CVP colony was stained negative for OCT4, a pluripotency marker, indicating that the colony did not arise from undifferentiated human ESCs. In addition, ISL1^+^ CVP colony was stained negative for HCN4, a recently reported FHF marker. Cell nuclei (blue) were stained with DAPI. Scale bar, 100 μm. (**g**) ISL1^+^ CVP clone transfected with siRNAs targeting *EDN1*, *EDNRA* or *EDNRB* showed ∼≥60% decrease in the number of ISL1^+^/CD24^−^/Pan-Neuronal^−^ cells compared with control RNAi, where a non-targeting siRNA was transfected. The CVP cells were transfected on alternate days with a final concentration of 60 nM of respective siRNAs. Cells were collected for flow cytometric analysis on day 10. Bars, s.d.; *n*=3 experiments. *P=0.0002 and **P=0.0001, evaluated by Student's *t*-test.

To validate our microarray data, we examined the expression of EDN1 in week 11 human fetal heart by immunocytochemistry, which revealed high expression of the growth factor in both OFT and right ventricle, regions derived from the SHF ISL1^+^ CVPs. Comparatively, much lower expression of the growth factor was observed in the left ventricle (Fig. 1a). Examination of the corresponding EDN1 receptors (EDNRA and EDNRB) in ISL1^+^ CVPs further suggested the utility of the EDN1 growth factor-receptor axis in the maintenance of CVPs multipotency (Fig. 1b), where the relative quantification of expressions of both receptors (EDNRA and EDNRB) is provided in Supplementary Fig. 1d. It is also noteworthy that human ESCs also express both EDN1 receptors (Supplementary Fig. 1e), albeit at a much lower level. Several studies have previously demonstrated that Notch signalling negatively regulates cardiac differentiation *in vivo* although the mechanism of its action remains largely unknown^19^,^20^. Consistently, we found that EDN1 activates Notch signalling effector gene targets such as *HEY1*, *HEY2*, *HEYL* and *HES1* in a dose-dependent manner (Supplementary Fig. 2a–c).

Next, we evaluated the utility of EDN1 in driving human ESC (ISL1 reporter cell line, H9G1) differentiation into SHF CVPs in a defined medium containing WNT3A, EDN1 or both. WNT3A was used as a comparison in our study, as multipotent ISL1^+^ CVPs were previously reported to be maintained and expanded on WNT3A over-expressing feeder cells^16^. Intriguingly, based on green fluorescent protein (GFP) expression, EDN1 was found to be inefficient in driving human ESC differentiation towards *ISL1*-expressing cells in the embryoid body assay although it was our top candidate ligand obtained from microarray analysis (Fig. 1c). More interestingly, the number of ISL1^*+*^-expressing cells (GFP^+^) was markedly reduced when both WNT3A and EDN1 were used in combination. However, through flow cytometric analysis, we determined that although fewer GFP^+^ cells were present in EDN1-containing medium, ∼70–80% of the GFP^+^ cells were ISL1^+^/CD24^−^/Pan-Neuronal^−^ (Fig. 1d). The overall number of ISL1^+^ CVPs generated in each condition however, remained relatively similar, suggesting that EDN1 restricts *ISL1* expression in cardiac mesoderm.

The multipotent ISL1^+^ CVPs can be clonally derived and expanded from human ESCs. Using a combination of EDN1 and WNT3A ligands^16^, single ISL1^+^ CVPs were sorted and expanded clonally, with a clonal efficiency of 1–2% (Fig. 1e and Supplementary Fig. 2d). In this study, ISL1^+^ CVP clones were successfully isolated and expanded from two human ESC ISL1 reporter lines, H9 and HUES3 (Supplementary Fig. 3), respectively. Immunostaining of the progenitor colonies demonstrated that they were positive for the SHF marker ISL1, but negative for the pluripotency marker OCT4, as well as the recently reported FHF marker, HCN4 (ref. 7; Fig. 1f).

To further validate the utility of EDN1 in clonal expansion of the multipotent CVPs, we adopted the RNA interference (RNAi) strategy to study the loss-of-function effects on these progenitors. EDN1 signalling was perturbed by two methods: siRNAs targeting *EDN1* to remove the endogenous growth factor and siRNAs targeting *EDNRA* and *EDNRB* to deplete the receptors that bind to EDN1. Analyses of the effects of RNAi on the maintenance of ISL1^+^ CVPs were performed by sorting for ISL1^+^/CD24^−^/Pan-Neuronal^−^ population. As expected, the percentage of cells that were ISL1^+^/CD24^−^/Pan-Neuronal^−^ were markedly reduced to <40% following *EDN1*, *EDNRA* or *EDNRB* RNAi treatment (Fig. 1g). Concomitantly, there was a decrease in the number of colonies formed once EDN1 axis was perturbed indicating that EDN1 maintains the ISL1^+^ cells in the progenitor cell state (Supplementary Fig. 4a). Quantitative PCR results confirmed that significant downregulation of *EDN1*, *EDNRA* and *EDNRB* transcripts (> 50%) was achieved by the respective siRNAs (Supplementary Fig. 4b–d). Together, these results clearly indicate the importance of EDN1 in the maintenance of ISL1^+^ CVPs, through autocrine and paracrine signalling. Consistently, *Edn-1* (also known as *ET1*) and *Ednra* knockout mice have been reported to exhibit cardiovascular malformations involving the SHF derivatives^21^,^22^,^23^. The mutant mice are characterized by interrupted aortic arch, tubular hypoplasia of the aortic arch, aberrant right subclavian artery and ventricular septal defect with abnormalities of the OFT.

### Long-term clonal expansion of multipotent SHF CVP

**Figure 2:**
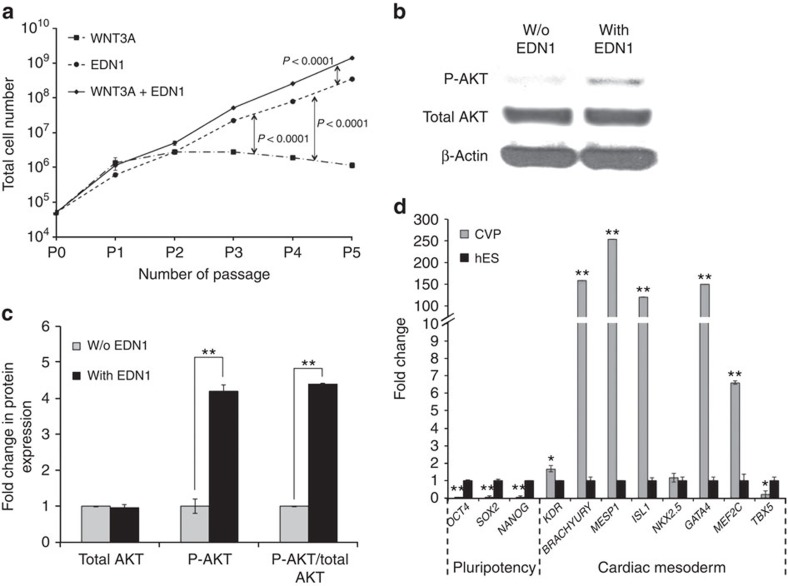
Endothelin-1 permits long-term clonal expansion of ISL1^+^ CVPs. (**a**) Growth profile of ISL1^+^ CVPs cultured over five passages in basal medium supplemented with 100 ng ml^−1^ WNT3A, 100 ng ml^−1^ EDN1 or both. The cells were passaged once they became confluent and continued to be cultured in their respective culture conditions. *n*=6 experiments. The *P* values between the experimental groups are as shown, evaluated by Student's *t*-test. (**b**,**c**) Immunoblot showing activation of Akt pathway in ISL1^+^ CVPs when cultured in the presence of EDN1. Refer to Supplementary Fig. 8 for full version of blots. Cell lysates were probed with antibodies against phospho-AKT, total AKT and β-actin (loading control). Densitometry of band intensities was performed using ImageJ software. **P<0.001, evaluated by Student's *t*-test. (**d**) Relative gene expression of pluripotent markers (*OCT4*, *SOX2* and *NANOG*) and cardiac mesoderm markers (*KDR*, *T-BRACHYURY*, *MESP1*, *ISL1*, *NKX2.5*, *GATA4*, *MEF2C* and *TBX5*) were assessed after ISL1^+^ CVP was cultured in defined medium supplemented with EDN1 and WNT3A for over 20 passages. *n*=3 experiments. *P<0.05 and **P=0.01, evaluated by Student's *t-*test. w/o, without.

To determine the expansion capacity of a single ISL1^+^ CVP cell, we explored the possibility of maintaining a continuous culture of ISL1^+^ CVP clones in defined media supplemented with EDN1, WNT3A or a combination of both for several passages (Fig. 2a). WNT3A over-expressing feeder was previously reported to support the expansion of ISL1^+^ SHF progenitors^16^. In the current study, long-term expansion of the cardiac progenitors in defined conditions revealed that cells cultured in the presence of WNT3A alone showed an initial increase in cell numbers (before P2), which declined thereafter, suggesting that WNT3A alone provides a signal for a short-term expansion of the CVPs, but not for their long-term maintenance. Once the progenitor cell differentiates, it becomes less proliferative resulting in a decline in cell number. In contrast, progenitors cultured in medium supplemented with only EDN1 illustrated an ∼7,000-fold increase in cell number over a period of five passages, while progenitors cultured in medium containing both EDN1 and WNT3A showed an increase of ∼28,000-fold in cell number during the same period (Fig. 2a), suggesting a synergistic effect between WNT3A and EDN1. The proliferative effect of EDN1 was illustrated through an increase in phosphorylated AKT levels in CVPs, with a fivefold increase in phosphorylated AKT/total AKT ratio as compared with control (Fig. 2b,c). The cell state of these ISL1^+^ CVPs was validated with quantitative PCR, which showed high expression of key cardiac mesodermal markers such as *ISL1*, *MESP1*, *GATA4*, *MEF2C* and *T-BRACHYURY* (Fig. 2d). It is noteworthy that at present, the expanded clones have been maintained in feeder-free conditions using a chemically defined medium for over 30 passages without losing their CVP identity. Taken together, these results point towards the role of EDN1 in maintaining the multipotency of CVPs, while WNT3A primarily functions to enhance its proliferative capability.

### ISL1^+^ CVP differentiates into cardiac derivatives *in vitro*

**Figure 3:**
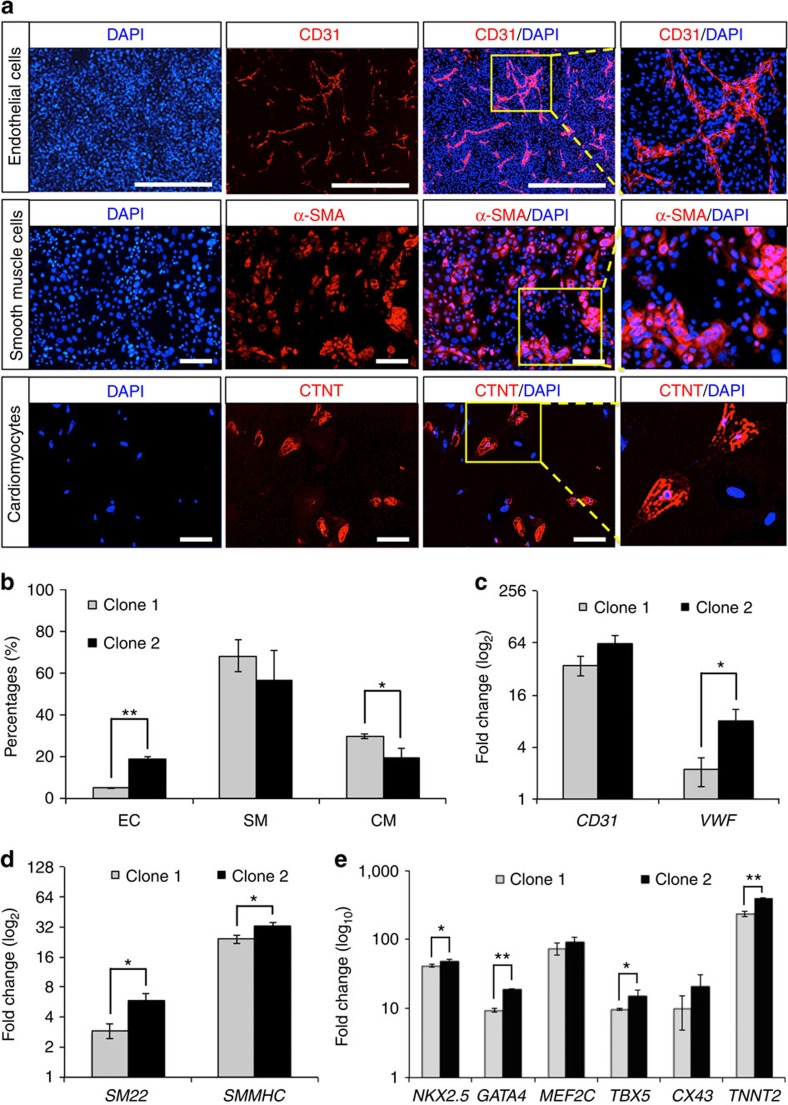
*In vitro* differentiation of ISL1^+^ CVP clones into cardiomyocytes, as well as endothelial and smooth muscle cells. Undifferentiated ISL1^+^ CVP clones were maintained in medium supplemented with EDN1 and WNT3A. Differentiation of the CVPs into various cardiac lineages was achieved using protocols described under material and method. (**a**) Immunocytochemistry demonstrating the presence of endothelial cell (EC; CD31^+^), smooth muscle cell (SM; SMMHC^+^) and cardiomyocyte (CM; CTNT^+^) on differentiation with various differentiation media. EC was stained with mouse anti-CD31 primary antibody while SM and CM were stained with mouse anti-SMMHC and mouse anti-CTNT, respectively, at 1:100 dilutions. Cell nuclei (blue) were stained with 4,6-diamindino-2-phenylindole (DAPI). Scale bar, 100 μm. (**b**) Graphical representation illustrating differentiation efficiencies of ISL1^+^ CVP into various cardiac lineages. Flow cytometric analyses for EC (CD31^+^), SM (SMMHC^+^) and CM (CTNT^+^) differentiation were performed on two progenitor clones (clones 1 and 2). Bars, s.d. of *n*=3 experiments. *P<0.05 and **P<0.001, evaluated by Student's *t*-test. (**c**–**e**) Quantitative PCR confirming the expression of *CD31* and *VWF* (EC markers); *SM22* and *SMMHC* (SM markers); and *NKX2.5*, *GATA4*, *MEF2C*, *TBX5*, *CX43* and *TNNT2* (CM markers) when ISL1^+^ CVP clones were differentiated into the various cardiac lineages. Gene expressions were normalized to the respective undifferentiated ISL1^+^ CVP clones. *n*=3 experiments. *P<0.05 and **P<0.01, evaluated by Student's *t*-test.

Thus far, we have identified EDN1 as a potent factor to clonally expand SHF ISL1^+^ CVPs. Because this progenitor pool holds great clinical potential, we wanted to investigate if it also possesses the hallmark of a multipotent progenitor, that is, the ability to differentiate into different distinct cardiac lineages. We assessed two ISL1^+^ CVP clones in generating cardiac lineages such as cardiomyocytes, endothelial cells and smooth muscle cells *in vitro* in accordance to published protocols^24^,^25^. Differentiation of CVPs to endothelial cells was first observed on day 3 and typically gave rise to 5–15% endothelial cells by day 7 (Fig. 3a,b), while specification towards cardiomyocytes (20–30%) and smooth muscle cells (50–70%) took 1–2 weeks. Contraction of clonally derived cardiomyocytes typically occurs 3–4 weeks after differentiation (Supplementary Movie 1). Identities of the differentiated cell types obtained from ISL1^+^ CVP clones were further confirmed by immunocytochemistry. Representative staining for endothelial cells, smooth muscle cells and cardiomyocytes were demonstrated by the presence of CD31, smooth myosin heavy chain (SMMHC) and cardiac troponin T (CTNT), respectively (Fig. 3a). Quantitative PCR of markers specific for the various cardiac lineages (endothelial cell: *CD31* and *VWF*; smooth muscle cell: *SM22* and *SMMHC*; cardiomyocyte: *NKX2.5*, *GATA4*, *MEF2C*, *TBX5*, *CX43* and *TNNT2*) further validated the expression of these genes (Fig. 3c–e).

### ISL1^+^ CVP primarily generate vasculature *in vivo*

The establishment of self-organizing organoids in *ex vivo* culture has become a new paradigm to evaluate differentiation potential of tissue-specific stem cells. Being a committed CVP, we envisage the CVPs to differentiate and self-organize into tissue organoids in cultures, similar to recent studies demonstrating formation of organoids obtained from kidney^26^,^27^, optic cup^28^, intestine^29^, stomach^30^, liver^31^, cerebral cortex^32^ and pancreas^11^,^33^. However, attempts to generate three-dimensional cardiac structures *in vitro* proved to be futile, possibly due to a lack of mesenchymal interactions or extracellular matrix reported to be important for tissue organogenesis and regeneration^34^,^35^,^36^. To circumvent this limitation, we injected CVPs (2 × 10^6^ cells) into the heart after induction of myocardial infarction, as well as the kidney capsules of NOD.Cg-Prkdcscid Il2rgtm1Wjl/SzJ (NOD/SCID) mice to assess for long-term engraftment, differentiation and self-assembly capabilities of CVPs *in vivo*.

**Figure 4:**
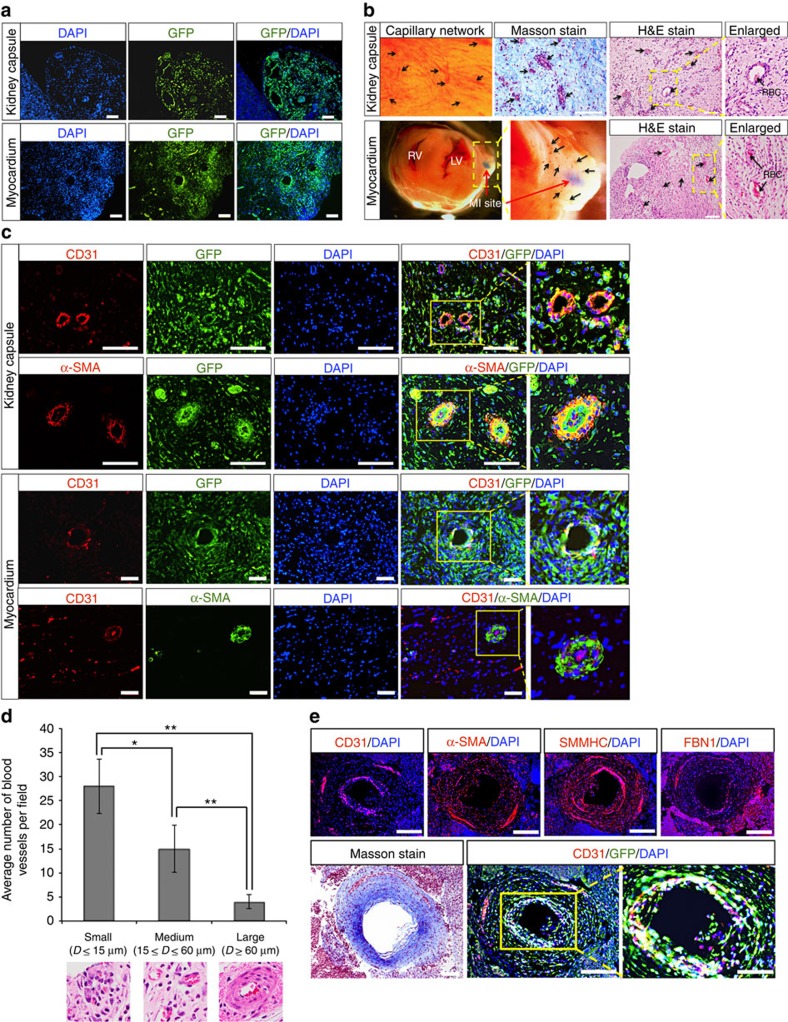
ISL1^+^ CVP differentiates and forms human–mouse chimeric circulatory system *in vivo*. (**a**) Xenografts stained positive for GFP present in kidney capsule and adult mouse heart after myocardial infarction (MI), demonstrating the engraftment of ISL1^+^ CVP-derived cells 3 weeks post transplantation. Cell nuclei (blue) were stained with 4,6-diamindino-2-phenylindole (DAPI). Scale bar, 100 μm. (**b**) Representative images of xenograft showing engraftment of ISL1^+^ CVP-derived differentiated cells in the kidney capsule and myocardium. Vasculature network could be seen from the surface of the xenograft (kidney capsule), with numerous blood vessels carrying red blood cells (RBCs) as shown in the haematoxylin and eosin (H&E) and Masson stains (indicated with black arrows). Site of MI is marked by red arrow (lower panel). Connective tissues revealed by Masson stain (stained blue) were observed throughout the xenograft. Enlarged images show functional blood vessels carrying blood cells. RV, right ventricle; LV, left ventricle. Scale bar, 100 μm. (**c**) Representative immunofluorescent image of muscularized blood vessels in xenografts (both kidney capsule and myocardium). Rings of endothelial cells were stained positive for CD31 (stained red). The arteries were surrounded by additional ring of smooth muscle cells that were stained positive for α-SMA. Cell nuclei (blue) were stained with DAPI. Scale bar, 50 μm. (**d**) Graphical representation of the numbers and sizes of blood vessels observed in the xenograft per field (transplantation into myocardium after MI). Results were tabulated from 10 random fields of H&E stains of the xenografts. Bars, s.d. of *n*=3 sections. *D*, diameter. *P<0.05 and **P<0.001, evaluated by Student's *t-*test. (**e**) Large coronary-like artery developed from the transplanted ISL1^+^ CVP in the kidney capsule. Immunofluorescent staining of the section revealed an inner layer of endothelial cells (stained positive for CD31), which is surrounded by a thick layer of smooth muscle cells that were stained positive for both SMMHC and α-SMA. Connective tissue surrounding the artery was further depicted by the presence of fibrillin-1 (FBN1) as well as Masson stain (blue). Cell nuclei (blue) were stained with DAPI. Scale bar, 100 μm. Scale bar in enlarged image, 50 μm.

Evidently, engraftment of the injected cells was achieved since GFP-labelled cells were present in both the kidney capsule and in the myocardium 3 weeks post transplantation (Fig. 4a). Haematoxylin and eosin, as well as Masson staining revealed that the xenograft in the kidney capsule comprised largely of pericytes, which belongs to the same cell lineage as vascular smooth muscle cells^37^,^38^,^39^,^40^, and connective tissues with numerous perforating functional blood vessels carrying blood cells (Fig. 4b). Similarly, formation of numerous GFP-labelled blood vessels that are anastomosed to host circulatory system (carrying blood cells) was also observed at the site of myocardial infarction, where the CVPs were transplanted (Fig. 4b and Supplementary Fig. 5). Interestingly, while the CVPs gave rise to all three cardiac lineages (endothelial cell, cardiomyocyte and smooth muscle cell) *in vitro* on various differentiation stimuli, our transplantation results revealed that only early passages of CVPs (<passage 10) can give rise to cardiomyocytes, in addition to endothelial and smooth muscle cells *in vivo* (Supplementary Fig. 6). It was observed that CVPs of later passages (>passage 20) almost exclusively contribute to vasculogenic lineages regardless of the sites of transplantation as evident by CD31, SMMHC and α-smooth muscle actin (α-SMA) staining (Fig. 4c,e). While the effects of continuous passaging on restricting lineage potential of the CVPs remained elusive, such restriction on differentiation potentials have been previously reported in other studies involving long-term expansion of human mesenchymal stem cells^41^,^42^ and human fetal neural stem cells^43^. Consistently, earlier studies have demonstrated that depletion of Edn-1 (ET1) in knockout mouse models resulted in severe cardiovascular malformations mainly involving vasculature formation in the developing heart^21^,^22^,^23^.

Close examination of the blood vessels formed revealed rings of α-SMA-positive cells surrounding the endothelial cells, resembling those of coronary arteries (Fig. 4c). Larger arteries (diameter ≥15 μm) were also present, albeit at a much lower frequency (Fig. 4d). These large arteries were found to possess an inner layer of CD31-positive cells that was surrounded by a thick layer of cells, which were stained positive for SMMHC, α-SMA and FBN1 (Fig. 4e). Through Masson staining, a ring of connective tissue and matrix was also found to be present throughout the thick wall of the larger coronary-like arteries (diameter ≥60 μm). A summary of the various types of blood vessels, based on their diameter sizes, and the frequency of their occurrence in xenografts are tabulated in Fig. 4d (injection into the myocardium) and Supplementary Fig. 7a (injection into the kidney capsule). Collectively, these results suggest that the CVPs possess the intrinsic ability to differentiate and self-organize into blood vessels *in vivo*, a feat not achievable by simply injecting a mixture of endothelial and smooth muscle cells into the kidney capsule or myocardium.

The propensities of these transplanted cells to form teratomas *in vivo* were also assessed through long-term transplantation assay. As expected, large teratomas were formed from kidney capsule injected with human ESCs (H9G1) after 10 weeks (Supplementary Fig. 7b). On the contrary, H9G1-derived CVPs injected into the kidney capsule were devoid of endoderm- and ectoderm-derived tissues, and only contained mesoderm-derived structures (Supplementary Fig. 7c).

## Discussion

The heart is an essential muscular organ that serves the primary role of maintaining the circulatory system in our body. Thus, the proper development and proliferation of the cardiac progenitors are critical requirements to ensure subsequent survival of embryos. The spontaneous formation of blood vessels in the xenografts resembles early stages of cardiogenesis during which key cardiac structures are quickly formed to ensure the proper development of a functional heart for the supply of nutrients and oxygen, as well as the removal of waste materials from the developing embryo, respectively.

While several recent studies have reported the derivation of coronary artery endothelial cells and the smooth muscle cells from the endocardium^44^ and epicardium^45^, respectively, we do not exclude the possibilities that vasculature formation could arise from cells originating from other regions of the heart. Lineage tracing experiments have shown that the ISL1^+^ CVPs give rise primarily to the OFT and the right ventricle^6^. As such, it is unlikely that the SHF ISL1^+^ CVP plays a significant role in vasculature formation in the endocardial wall particularly during rapid expansion of the compact myocardium. Instead, we hypothesize that the *in vivo* generation of *de novo* vasculature after cell injection resembles the early stages of heart development, in particular the formation of the proximal end of the coronary artery.

Evidently, this suggests that a single CVP cell possesses the ability to self-renew, differentiate into other cardiac lineages and self-assemble into functional cardiac-like tissues. Although examination of long-term transplanted xenografts revealed a lack of atrial- or ventricular-like structures, the xenografts were mostly packed with vasculatures that were supported by stromal elements and connective tissues such as collagen, fibronectin, elastin and so on. Occasionally, large arteries with thick layer of smooth muscle cells surrounding a thin inner ring of endothelial cells were found in the xenograft. The frequencies of these differentially sized arteries and capillaries present in the xenografts reflect an organized angiogenesis process, signifying the formation of a hierarchical vascular network in a developing organ^46^,^47^.

**Figure 5:**
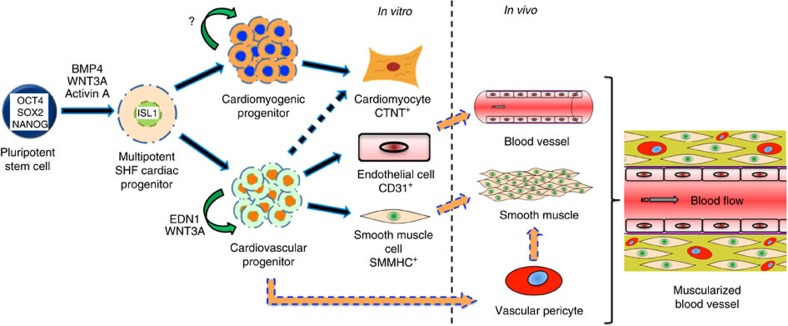
Schematic illustration of the importance of EDN1 in the maintenance of CVPs. We proposed that EDN1 promotes the clonal expansion of CVPs. Given the appropriate stimuli, CVPs were able to differentiate towards all three cardiac lineages (cardiomyocyte, smooth muscle cell and endothelial cell) *in vitro* (<10 passages) while almost exclusively contributed to vascular lineages *in vivo* (>20 passages). This suggests that EDN1 primes the CVP towards a vascular lineage while WNT3A functions primarily to enhance their proliferation.

In summary, our study identifies EDN1 as a key component, acting through both autocrine and paracrine means to maintain and expand clonal ISL1^+^ CVPs. These cultured CVPs were eventually shown to differentiate primarily into endothelial and smooth muscle cell lineages, forming a functional human–mouse chimeric circulatory system *in vivo* (summarized in Fig. 5). The isolation, culture and expansion of ISL1^+^ CVP clones demonstrates the intrinsic autonomous nature of this progenitor cell to differentiate and self-organize into functional vasculatures. The ability to generate blood vessels from CVPs henceforth presents the potential to model developmental diseases or degenerative conditions involving the heart and circulatory system by introducing disease mutations using CRISPR technology^48^. Moving forward, culturing CVPs in defined conditions thus opens avenues for the development of regenerative therapeutics and tissue engineering approaches to generate vascularized cardiac patches or blood vessels for cell-based therapies^49^.

## Methods

### Cell culture of H9G1 human ES and H9G1-ISL1^+^ SHF CVPs

The human ESC line (H9G1) and HUES3 ISL1-RFP reporter cell lines, previously generated in our laboratory^4^, were cultured in feeder-free condition on Matrigel (BD, Franklin Lakes, NJ). The cells were maintained in mTeSR1 medium (Stemcell Technologies). For culture of ISL1^+^ CVPs, a basal medium (CGM) containing 35% complete IMDM/65% DMEM–Ham F-12 mix containing 2% B27, 2% BSA Fraction V, 0.1 mmol l^−1^ 2-mercaptoethanol, 4 ng ml^−1^ epidermal growth factor, 10 ng ml^−1^ basic fibroblast growth factor, 40 nmol l^−1^ cardiotrophin-1, 40 nmol l^−1^ thrombin, 1% Pen/Strep and 1% L-glutamine. Endothelin-1 and WNT3A were supplemented fresh into the medium.

### Gene knockdown using siRNA in H9G1-ISL1^+^ SHF progenitor

Silencer select pre-designed siRNAs targeting human *EDN1*, *EDNRA*, *EDNRA* and control siRNA (Ambion) were used for transfection into ISL1^+^ CVPs using Lipofectamine RNAi MAX Transfection Reagent (Invitrogen), according to the manufacturer's recommendation. The siRNA sequences are provided in Supplementary Data 8. Briefly, 60 nM of siRNA was used for each transfection into 1.0 × 10^5^ cells in suspension, and subsequently plated onto a 12-well tissue culture plate. Repeated transfection was performed with respective siRNAs every alternate day. The cells were collected after 10 days of culture for flow cytometric and quantitative real-time PCR (qPCR) analysis.

### Human ES differentiation and clonal isolation of ISL1^+^ CVP

The human ES (H9G1) and HUES3 ISL1-RFP reporter cell lines were differentiated to embryoid bodies as described by Bu *et al.*^4^ Briefly, human ESCs were treated with collagenase (1 mg ml^−1^; Gibco), and then suspended in differentiation medium containing BMP4 (20 ng ml^−1^), activin A (10 ng ml^−1^) and WNT3A (100 ng ml^−1^; all from R&D Systems) before transferring to six-well ultra low cluster plates (Costar), and cultured at 37 °C. ISL1-expressing cells (GFP- or RFP-positive) were typically observed on day 7 after differentiation. The embryoid bodies were dissociated with accutase (Life Technologies). Single-cell suspension was obtained, and the cells were stained with PE-conjugated mouse anti-CD24 (BD Bioscience) and mouse anti-human Pan-Neuronal marker (Millipore) at concentration of 1:100 dilution, followed by donkey anti-mouse IgG Alexa 647 at 1:1,000 dilution (Invitrogen). Isolation of ISL^+^ CVP was achieved by fluorescence-activated cell sorting for GFP^+^/CD24^−^/Pan-Neuronal^−^ or RFP^+^/CD24^−^/Pan-Neuronal^−^ cells. Single-sorted cells were seeded onto irradiated feeder layer (MEF) and cultured with CGM medium supplemented with EDN1 and WNT3A.

### RNA extraction and cRNA synthesis

For cultured cell samples, 2 × 10^6^ cells were collected and lysed in 800 μl of TRIzol reagent (Invitrogen). The samples were allowed to stand for 5 min at room temperature, after which 160 μl of chloroform was added to allow for phase separation by centrifugation at 12,000*g* for 15 min at 4 °C. Following that, the aqueous phase was transferred to a fresh tube, and equal volume of isopropanol was added and mixed. RNA samples were allowed to precipitate at room temperature for another 10 min. The precipitated RNA samples were pelleted by centrifugation at 12,000*g* for 15 min at 4 °C. Five hundred nanogram of total RNA from each sample were used to generate cRNA using the Illumina Totalprep RNA Amplification Kit (Ambion) according to the manufacturer's recommendations. Briefly, RNA samples were reverse transcribed to synthesize first strand cDNA by mixing 11 μl of RNA samples (500 ng) with 9 μl of reverse transcription master mix and incubated at 42 °C for 2 h. An additional 80 μl of second strand master mix was added into the sample and incubated for 2 h at 16 °C to generate the second strand cDNA. Purification of cDNA was achieved by adding 250 μl of cDNA-binding buffer to each sample. The mixture was passed through a cDNA filter cartridge and washed with 500 μl of wash buffer. cDNA was eluted with 20 μl of 55 °C nuclease-free water. *In vitro* transcription to synthesize cRNA was achieved by adding 7.5 μl of IVT master mix to each cDNA sample, and incubated for 14 h at 37 °C. A volume of 75 μl of nuclease-free water was added to each sample to stop the reaction. Purified cRNA samples were subsequently obtained by adding 350 μl of cRNA-binding buffer to each sample, followed by an additional 250 μl of 100% ethanol to precipitate the cRNA samples. The mixture was passed through cRNA filter cartridge, and washed with 650 μl of wash buffer. Purified cRNA samples were eventually eluted with 100 μl of 55 °C nuclease-free water.

### Reverse transcription and qPCR

RNA samples (500 ng) were reverse transcribed to obtain cDNA using the iScript cDNA Synthesis kit (Bio-Rad). Primer sequences are provided in Supplementary Data 9. Quantitative PCR analyses were performed using the ABI Viia7 Real Time PCR System. The threshold cycle (Ct) was determined to be ≥35. Each experiment was repeated at least twice. S.d.'s of the means in qPCR experiments were obtained from three independent experiments.

### Microarray analysis

Microarray study was conducted using HumanHT-12 v4 BeadChip (Illumina). The microarray data have been deposited in GEO under accession code GSE75985. Hybridization and scanning of the BeadChip were performed using the Illumina BeadArray Reader. All gene expression data were first subtracted from the background, and then normalized using the cross-correlation.

### Immunocytochemistry

Immunocytochemical analysis was performed using the respective antibodies: mouse anti-human CD31 monoclonal antibody (AbD Serotec; 1:100); mouse anti-ISL1 monoclonal antibody (Hybridoma Bank; 1:200); mouse anti-CTNT polyclonal antibody (Thermo Scientific; 1:250); rabbit anti-SMMHC polyclonal antibody (Biomedical Technologies; 1:200); and mouse anti-human SMA monoclonal antibody (DAKO; 1:250). Cells were first collected and washed once with PBS. Fixation of cells was achieved with 4% paraformaldehyde for 30 min, followed by treatment with 0.3% Triton X to permeabilize cells, if necessary. The cells were then blocked with PBS containing 5% fetal bovine serum and 1% BSA for 30 min at room temperature. Primary antibodies were added at recommended dilutions and incubated for 1 h at room temperature. After washing, the cells were incubated for another 45 min at room temperature without light exposure with either 1:1,000 diluted Alexa Fluor 488 or Alexa Fluor 594 secondary antibodies. Nuclei were counterstained with 4,6-diamindino-2-phenylindole. The cells were observed under a fluorescent microscope (Nikon TS-100).

### Western blot

CVPs were cultured in basal medium in the presence or absence of endothelin-1 growth factor for 5 days. For the preparation of cell lysates, cell pellets were incubated in RIPA lysis buffer (1% Nonidet P-40, 0.5% deoxycholate, 5 M NaCl and 1 M Tris (pH 7.4)) for 10 min at 4 °C, and then centrifuged at 11,400*g* for 15 min at 4 °C. Quantification of protein extract was carried out using the Protein Assay (Bio-Rad) according to the manufacturer's instructions. Electrophoretic analysis was performed using 10–15% SDS–PAGE gel (Bio-Rad). Gels were blotted onto nitrocellulose membrane (Amersham Biosciences), which were then probed with rabbit anti-human AKT, anti Phospho-AKT (Ser473; Cell Signaling), mouse anti β-actin (Santa Cruz), rabbit anti-human HEY1 and HEY2 (both from GeneTex) and mouse anti α-tubulin (Santa Cruz) primary antibodies according to the manufacturer's instructions. Primary antibodies were detected with species-specific horseradish peroxidase-conjugated secondary antibodies (Santa Cruz) and ECL western blotting detection system (Amersham Biosciences).

### Transplantation of ISL1^+^ CVPs into mouse kidney capsule

NOD.Cg-Prkdcscid Il2rgtm1Wjl/SzJ (NOD/SCID) mouse strain harbouring a null mutation of the common cytokine receptor γ chain (Il2rgtm1Wjl) (NOD/SCID/IL2rγnull) from Jackson Laboratory (Bar Harbor, ME) was used for cell transplantation. All procedures were performed on male mice between 8 and 10 weeks of age. Briefly, mice were anaesthetized with 2.5% avertin, and 2 × 10^6^ ISL1^+^ CVPs were injected into the kidney capsule using an 18G tuohy epidural needle.

### Myocardial infarction and lectin perfusion

All surgical and experimental procedures with mice were done in accordance with European Community Council Directives and approved by the Stockholm's Norra Djurförsöketiska Nämnd (Stockholm, Sweden). Myocardial infarction was induced in 8-week-old male NOD.Cg-Prkdcscid Il2rgtm1Wjl/SzJ (NOD/SCID) mouse strain by permanent ligation of the left anterior descending, as previously described^35^. Briefly, the left thoracic region was shaved and sterilized. The animal was intubated, and positive pressure ventilation was provided by a ventilator, where the tidal volume and rate were determined by preprogrammed settings based on animal weight. The heart was exposed through a left thoracotomy (transverse incision was made between the fourth and fifth intercostal spaces) and a suture with a no. 4½ circle taper point needle was used to ligate the left anterior descending coronary artery. The thoracotomy and skin were sutured closed in layers, and the mouse was removed from ventilation when normal breathing was established. The procedure was completed in ∼30 min, after which the animal was placed in a heated holding cages for recovery.

Lectin perfusion was performed at least 1 month after grafting according to established protocol^50^. Briefly, tomato lectin from *Lycopersicon esculentum* (DL-1177, Vector laboratories) at a final concentration of 1 mg ml^−1^ was injected into the tail vein of anaesthetized mice. Lectin binds to the luminal surface of endothelial cells of human and mice blood vessels. Mice were killed 5 min later and the hearts were collected and fixed in 4% paraformaldehyde.

### Histopathology and immunochemical staining

Mice were killed either 3 weeks (short term) or 10 weeks (long term) post transplantation. Transplanted kidneys were collected and fixed in 4% paraformaldehyde. The tissues were processed with Tissue Processor (Leica Microsystems, Buffalo Grove, IL) and embedded in paraffin. Sections were cut at a 5-μm thickness, mounted on poly-lysine-coated slides (Thermal Fisher Scientific, Pittsburgh, PA), de-waxed, rehydrated and processed for haematoxylin and eosin an Masson staining according to standard protocols. For immunofluorescent staining, antigen retrieval was carried out using citrate buffer (10 mM citric acid, 0.05%, pH 6.0). Sections were then immersed for 1 h in blocking buffer from M.O.M basic kit (Vector Laboratories), then incubated in primary antibody (made up in M.O.M diluent buffer) at 4 °C overnight, followed by incubation in secondary antibody at 37 °C for 1 h. Rabbit anti-GFP polyclonal antibody (Abcam), mouse anti-human CD31 monoclonal antibody (AbD serotec), mouse anti-CTNT polyclonal antibody (Thermo Scientific), mouse anti-SMMHC polyclonal antibody (Biomedical Technologies), rabbit anti-human FBN1 polyclonal antibody (Abcam) and rabbit anti-GFP polyclonal antibody (Abcam) were used at 1:100 dilution. Secondary antibodies, donkey anti-rabbit or anti-mouse with different Alexa Fluor conjugations were purchased from Invitrogen and used at a 1:1,000 dilution. All sections were counterstained with 4,6-diamindino-2-phenylindole (blue). Sections were mounted with anti-fade reagent (Vectashield) and then viewed under a fluorescent microscope (Nikon TS-100).

### Statistics

qPCR values are expressed as mean±s.d. Results were tested for statistical significance using Student's *t*-test, two sided based on assumed normal distributions. *P* values <0.05 were considered statistically significant.

## Additional information

**How to cite this article:** Soh, B.-S. *et al.* Endothelin-1 supports clonal derivation and expansion of cardiovascular progenitors derived from human embryonic stem cells. *Nat. Commun.* 7:10774 doi: 10.1038/ncomms10774 (2016).

## Supplementary Material

Supplementary Figures Supplementary Figures 1-8

Supplementary Movie 1 Contracting cardiomyocyte video. The video illustrates the contraction of cardiomyocytes derived from a single colony of cardiovascular progenitor maintained in the presence of endothelin-1.

Supplementary Data 1 List of growth factor in wk 11 left atrium

Supplementary Data 2 List of growth factor in wk 11 left ventricle

Supplementary Data 3 List of growth factor in wk 11 right atrium

Supplementary Data 4 List of growth factor in wk 11 right ventricle

Supplementary Data 5 List of growth factor in wk 11 outflow tract

Supplementary Data 6 List of growth factor receptor in ISL1 positive cells (above 1.2 fold)

Supplementary Data 7 List of growth factor-receptor interaction pairs

Supplementary Data 8 siRNA sequences

Supplementary Data 9 List of primer sequences
