# Corrigendum: Endothelin-1 supports clonal derivation and expansion of cardiovascular progenitors derived from human embryonic stem cells

**DOI:** 10.1038/ncomms12118

**Published:** 2016-07-19

**Authors:** Boon-Seng Soh, Shi-Yan Ng, Hao Wu, Kristina Buac, Joo-Hye C. Park, Xiaojun Lian, Jiejia Xu, Kylie S Foo, Ulrika Felldin, Xiaobing He, Massimo Nichane, Henry Yang, Lie Bu, Ronald A Li, Bing Lim, Kenneth R Chien

Nature Communications 7: Article number: 1077410.1038/ncomms10774 (2016); Published: 03 08 2016; Updated: 07 19 2016.

The affiliation details for Boon-Seng Soh, Lei Bu and Ronald A. Li are incorrect in this Article. The correct addresses of these authors are listed below:

**Boon-Seng Soh**

Cardiovascular Research Center, Massachusetts General Hospital, 185 Cambridge Street, Boston, Massachusetts 02114, USA; Department of Stem Cell and Regenerative Biology, Harvard University, 7 Divinity Avenue, Cambridge, Massachusetts 02138, USA; Li Dak-Sum Research Centre-HKU-Karolinska Institutet Collaboration on Regenerative Medicine, University of Hong Kong, Hong Kong, China and Department of Cell and Molecular Biology and Medicine, Karolinska Institute, Stockholm S-171 77, Sweden.

**Lei Bu**

Cardiovascular Research Center, Massachusetts General Hospital, 185 Cambridge Street, Boston, Massachusetts 02114, USA; Department of Stem Cell and Regenerative Biology, Harvard University, 7 Divinity Avenue, Cambridge, Massachusetts 02138, USA and Li Dak-Sum Research Centre-HKU-Karolinska Institutet Collaboration on Regenerative Medicine, University of Hong Kong, Hong Kong, China.

**Ronald A. Li**

Li Dak-Sum Research Centre-HKU-Karolinska Institutet Collaboration on Regenerative Medicine, University of Hong Kong, Hong Kong, China.

