# Supplementary material for: Endothelin-1 supports clonal derivation and expansion of cardiovascular progenitors derived from human embryonic stem cells
Source: Nat Commun. 2016 Mar 8;7:10774. doi: 10.1038/ncomms10774 (PMC4786749; doi:10.1038/ncomms10774)
Supplement: Supplementary Figures — 1-8 [file ncomms10774-s1.pdf]

SUPPLEMENTARY FIGURES AND FIGURE LEGENDS

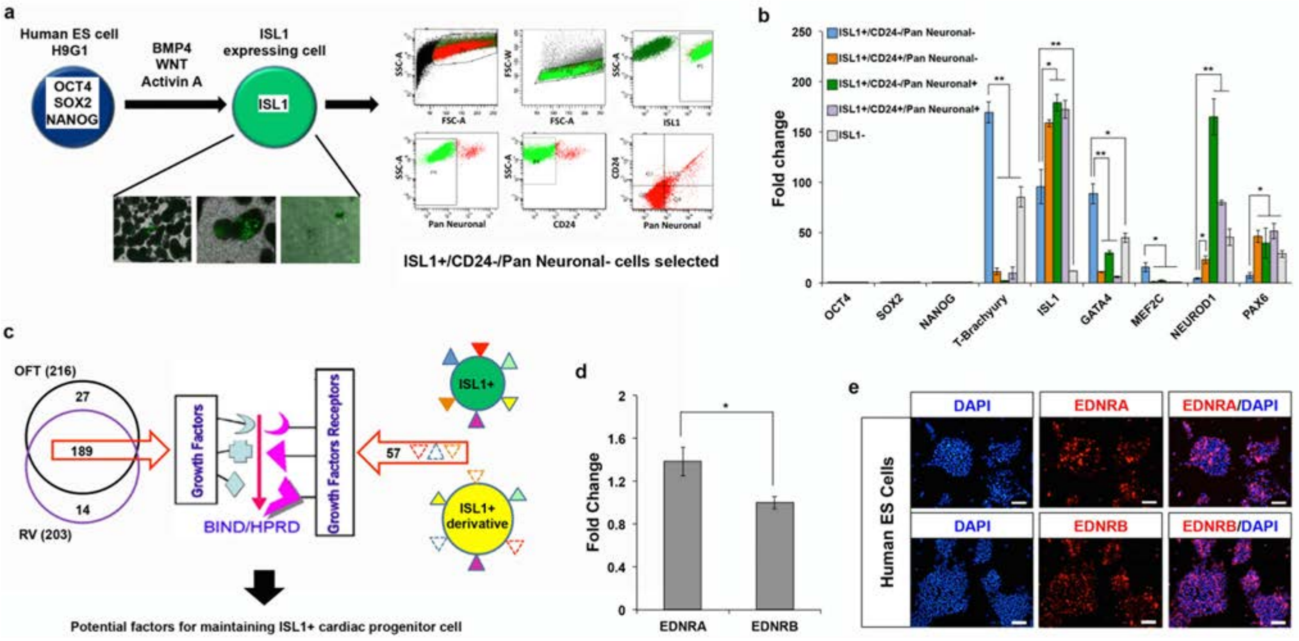

Supplementary Figure 1. Methodology and bio-computational analysis to obtain useful ligand-receptor interactions utilized by ISL1$^+$ CVP to maintain multipotency.

a) Schema for directed differentiation of ISL1$^+$ cardiovascular progenitors using the human ISL1-GFP (H9G1) and HUES3 ISL1-RFP reporter cell lines. Cells were sorted for ISL1$^+$/Pan Neuronal$^-$/ CD24$^-$ and seeded onto feeders for clonal expansion.

b) Quantitative PCR of pluripotent markers (*OCT4*, *SOX2*, *NANOG*) and cardiac mesoderm markers (*T-BRACHYURY*, *ISL1*, *GATA4*, *MEF2C*) and other lineage markers such as *NEUROD1* (neuroectoderm) and *PAX6* (pancreatic islet) were assessed on various ISL1$^+$ and ISL1$^-$ cell sorted fractions. Gene expressions were normalized to undifferentiated human ES cells. n = 3 experiments. * indicates $p < 0.05$ and ** indicates $p < 0.001$, evaluated by Student's t-test.

c) Schema illustrating the approach to identify potential ligand-receptor interaction pairs in the maintenance of multipotent ISL1$^+$ CVPs. Using interaction data from BIND and HPRD, 8 potential growth factors functioning in 11 interaction pairs were identified.

d) Graphical representation of the expressions of EDNRA and EDNRB receptors on ISL1$^+$ CVPs. Image analysis was performed using Columbus (Perkin Elmer). n = 6 images. * indicates $p < 0.05$, evaluated by Student's t-test.

e) Immunocytochemistry demonstrating expressions of EDNRA and EDNRB cell surface receptors (stained red) for EDN1 in human ES cells. Cell nuclei (blue) were stained with DAPI. Scale bar, 50 µm.

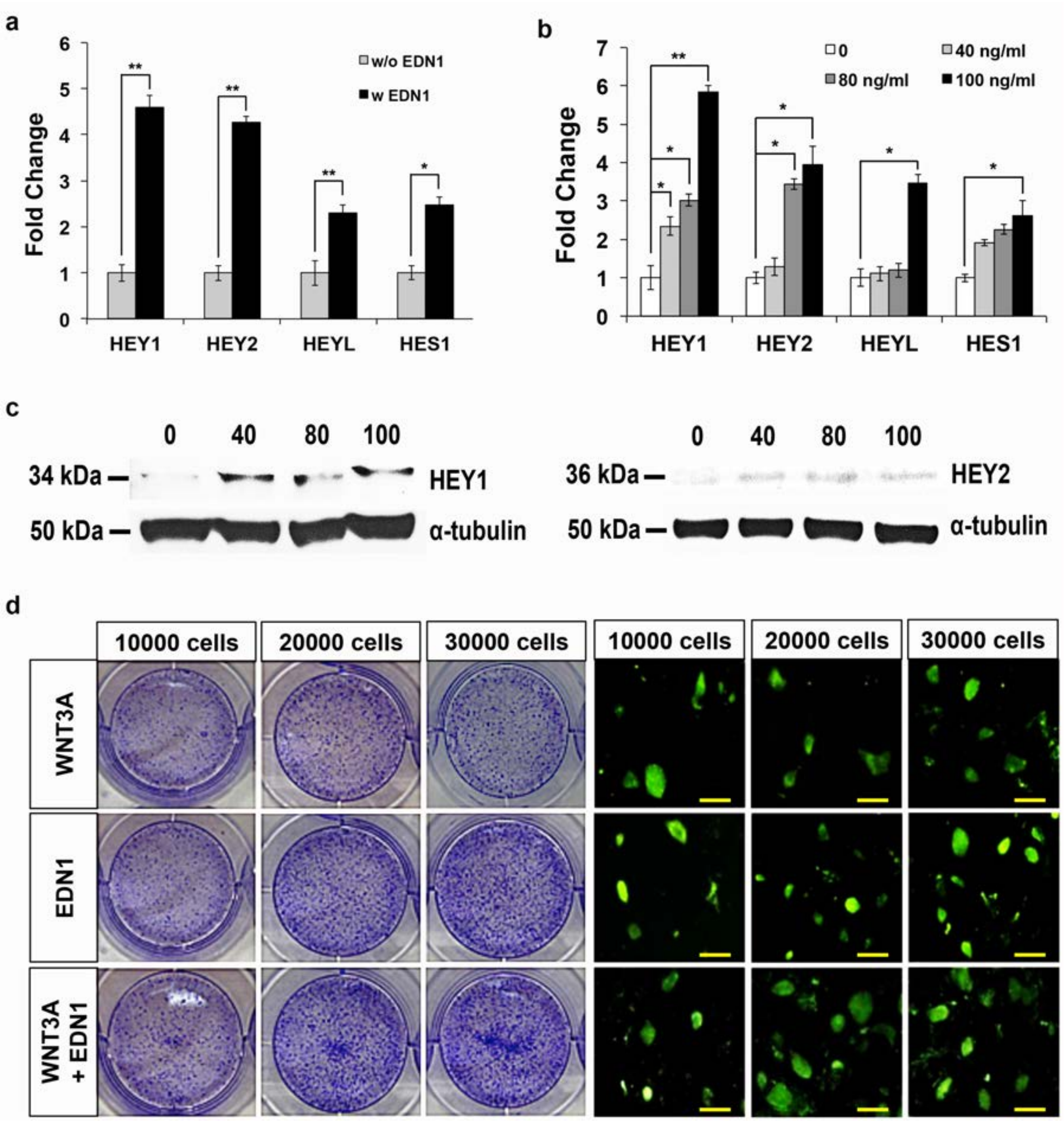

Supplementary Figure 2. Endothelin-1 activates Notch signaling in a dose dependent manner to maintain multipotency of $ISL1^+$ cardiovascular progenitor cell.

a-b) Quantitative PCR illustrating the activation of Notch signaling effector genes (*HEY1*, *HEY2*, *HEYL* and *HES1*) in $ISL1^+$ CVPs in a dose dependent manner when cultured in the presence of EDN1. n = 3 experiments. * indicates $p < 0.05$ and ** indicates $p < 0.01$, evaluated by Student's t-test.

c) Immunoblots showing expression of Notch signaling effector proteins, HEY1 and HEY2 in $ISL1^+$ CVPs in a dose dependent manner when cultured in the presence of EDN1 (0, 40, 80 and 100ng/ml). Cell lysates were probed with antibodies against HEY1 and HEY2, and α-Tubulin (loading control).

d) Colony forming efficiency of the $ISL1^+$ CVPs was assessed by seeding single progenitor cells on MEF feeders at various cell densities (10000, 20000 and 30000 cells) per well in six-well plates. GFP^+ colonies were visible after day 7 and were subsequently stained with crystal violet for quantification. Scale bar, 500 μm.

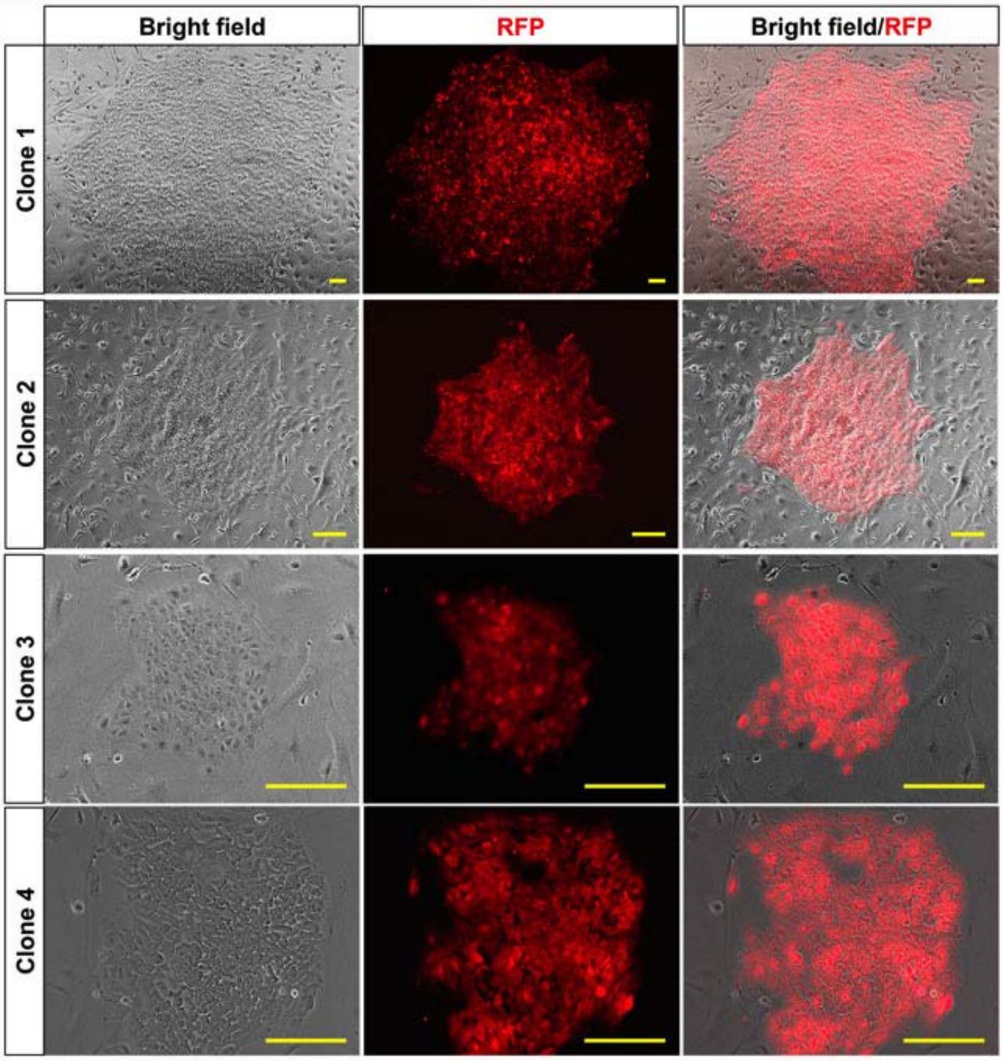

Supplementary Figure 3. Clonal isolation and expansion of multipotent ISL1$^+$ CVPs from HUES3 ISL1-RFP reporter cell line [1]. Representative images of four ISL1$^+$ CVP clones (expressing RFP) were shown. Scale bar, 100 μm.

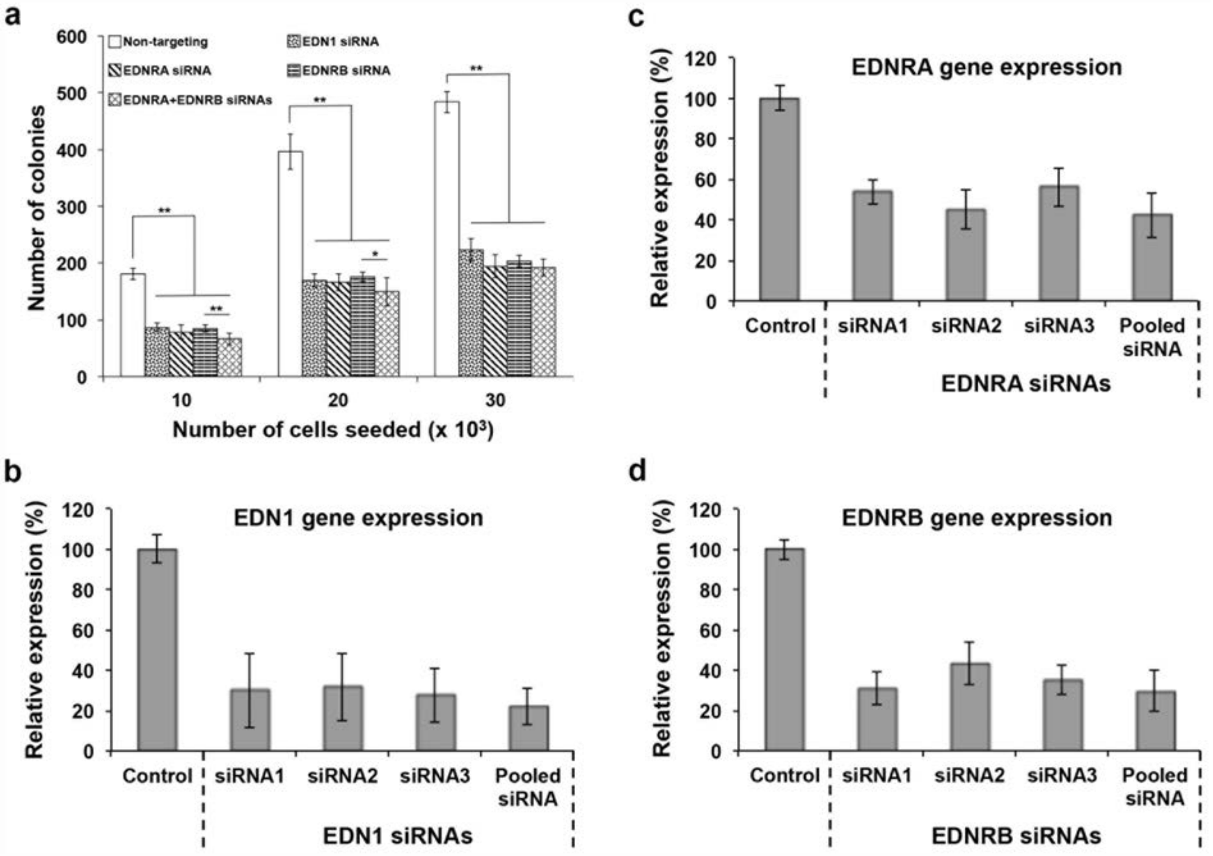

Supplementary Figure 4. Endothelin-1 pathway enhances colony forming efficiency of ISL1$^+$ CVPs.

a) Colony forming efficiency of the ISL1$^+$ CVPs was assessed after siRNA knockdown of genes encoding for EDN1, EDNRA, EDNRB, or both EDNRA and EDNRB. A non-targeting siRNA was used as control. The CVP cells were transfected on alternate days with a final concentration of 60 nM of respective siRNAs. Single progenitor cells were seeded on MEF feeders at various cell densities (10000, 20000 and 30000 cells) per well in six-well plates. Colonies were visible after day 7 and were subsequently stained with crystal violet for quantification. Bars, s.d., n = 3 experiments. * indicates $p < 0.05$ and ** indicates $p < 0.001$.

b-d) Relative gene expressions of *EDN1*, *EDNRA* and *EDNRB* in cardiovascular progenitors 10 days after transfection with respective siRNAs. The results were normalized to scrambled transfection. Bars, s.d., n = 4 experiments.

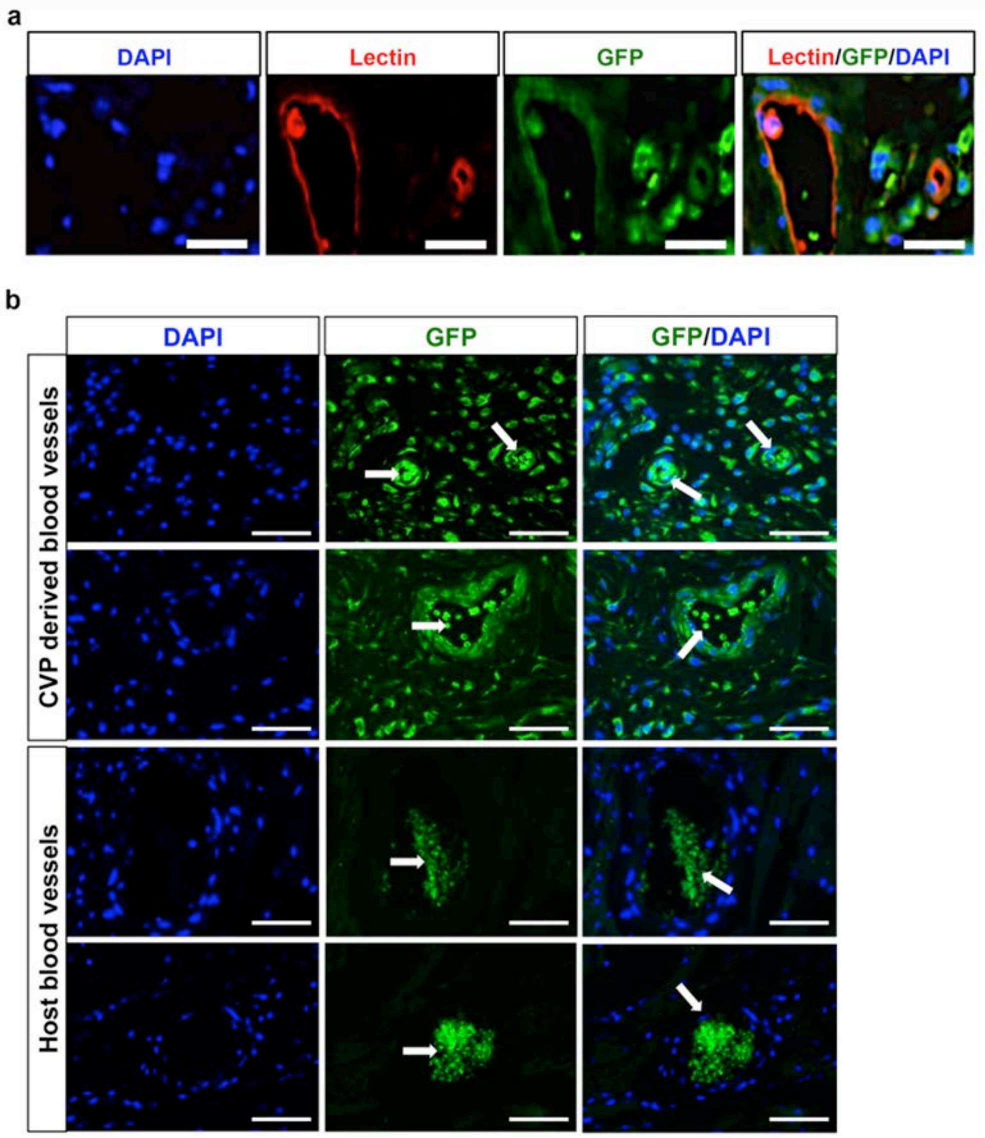

Supplementary Figure 5. ISL1$^+$ CVPs form functional human-mouse chimeric circulatory system *in vivo*.

a) Representative images of xenograft derived from ISL1$^+$ CVP differentiated cells in the myocardium. Infarcted heart transplanted with ISL1$^+$ CVPs was perfused with fluorescent labeled lectin (red), combined with immunofluorescent labeling of GFP (green). Cell nuclei (blue) were stained with DAPI. Scale bar, 50 µm.

b) Representative immunofluorescent images of patent GFP$^+$ blood vessels in xenografts (myocardium). GFP$^+$ blood vessels were shown to carry auto-fluorescing red blood cells (white arrows) indicating that the human ISL1$^+$ CVPs derived blood vessels were connected to the mouse circulatory system. In contrast, although the host (mouse) blood vessels in the infarcted heart carry red blood cells (green auto-fluorescence), they are not GFP labeled (not derived from ISL1$^+$ CVP). Cell nuclei (blue) were stained with DAPI. Scale bar, 50 µm.

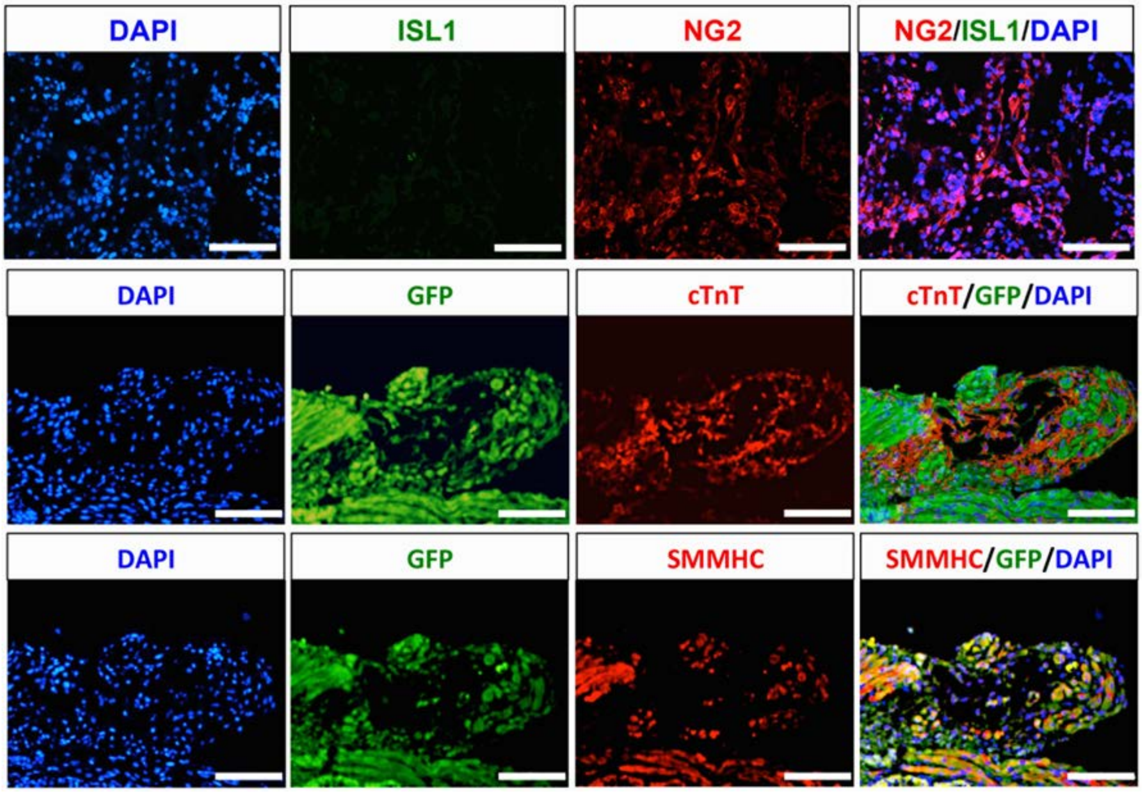

Supplementary Figure 6. Early passages of $ISL1^+$ progenitor clones gave rise to cardiomyocyte differentiation *in vivo*. Representative immunofluorescence images of xenograft demonstrating the presence of differentiated cardiac cell types. The xenograft was obtained by transplanting $ISL1^+$ CVPs (Passage <10) into the kidney capsule. ISL1 staining of the xenograft showed absence of undifferentiated $ISL1^+$ CVPs. Pericytes, which arise from vascular smooth muscle cells were stained positive for NG2 (red), while the cardiomyocytes and smooth muscle cells were stained positive for cTnT (red) and SMMHC (red), respectively, with Alexa 594 secondary antibody while GFP was stained green. Cell nuclei (blue) were stained with DAPI. Scale bar, 100µm.

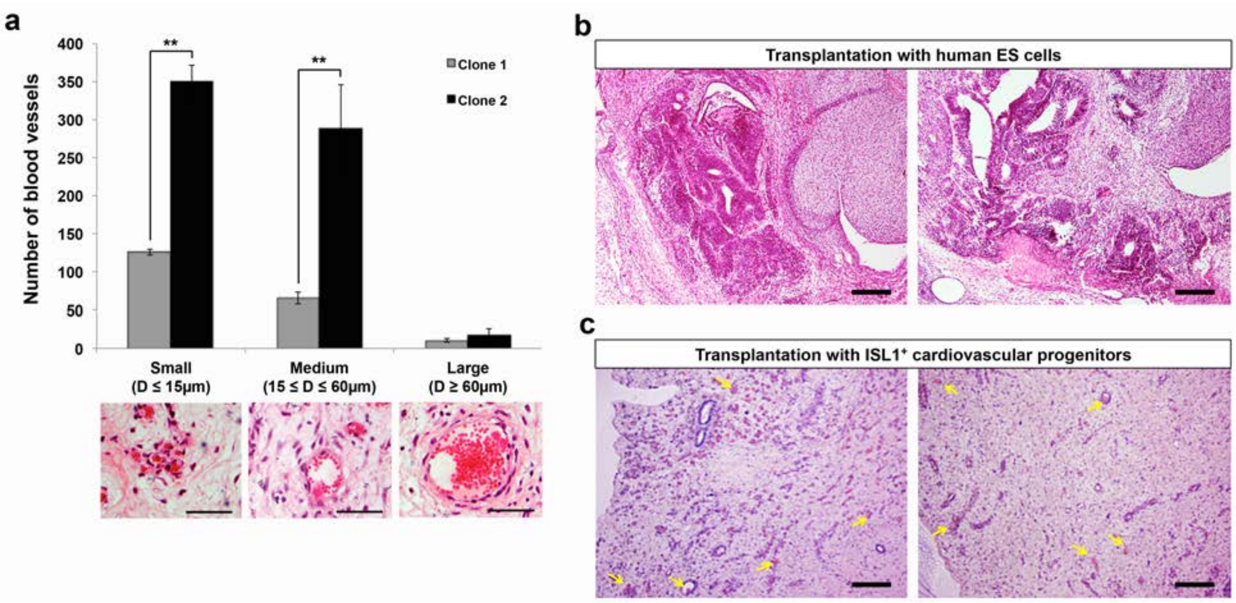

Supplementary Figure 7. Long-term transplantation of ISL1^{+} cardiovascular progenitor does not form teratomas *in vivo*.

a) Graphical representation of the total numbers and sizes of blood vessels observed in the xenograft (kidney capsule) obtained from two ISL1^{+} CVP clones. Results were tabulated from 25 random fields of H&E stains of the xenografts. Bars, standard deviation of n = 3 sections. D; diameter. ** indicates $p \leq 0.001$, evaluated by Student's t-test. Scale bars, 50 µm.

(**b-c**) Representative images of H&E staining of xenografts derived from transplanting (b) human ESCs and (c) ISL1^{+} CVPs 10 weeks post-transplantation. Note the presence of endoderm-, mesoderm- and ectoderm-derivatives in teratoma formed from transplanting undifferentiated human ESCs (H9G1 cell line) (b). Yellow arrows (c) depict the presence of red blood cells, indicating the presence of functional blood vessels in the xenograft derived from transplanting ISL1^{+} CVPs. Scale bars, 200 µm.

2b

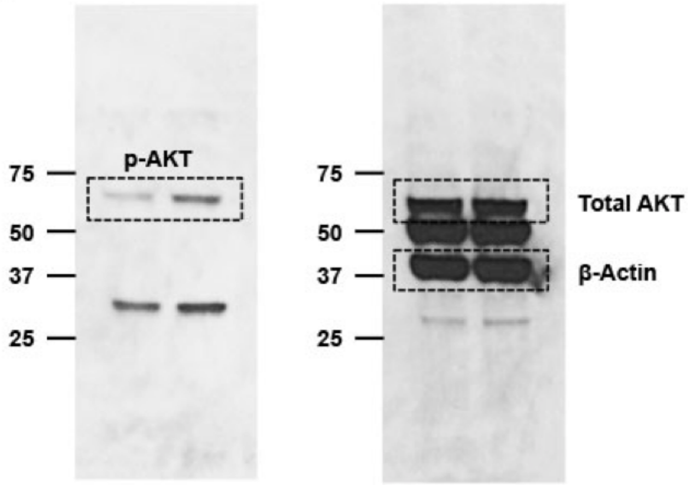

Supplementary Figure 8. Full versions of western blots. Expanded and uncropped versions of representative western blots shown in main figures. All numbers are ladder sizes in kDa. Panels are labeled with the corresponding main figure. Individual blots within a panel are labeled with the antibody for cross-reference with the figure.

Reference

1. Bu, L. et al. Human ISL1 heart progenitors generate diverse multipotent cardiovascular cell lineages. *Nature* **460**, 113-117 (2009).
